# Burnout levels and associated factors among physicians and dentists in Türkiye

**DOI:** 10.1097/MD.0000000000048108

**Published:** 2026-03-20

**Authors:** Mehmet Yildiz, Muhammet Raşit Aydin, Abdülkadir Aydin

**Affiliations:** a Department of Family Medicine, Giresun Provincial Health Directorate, Bulancak Sehit Er Enver Erdogan Family Health Center, Giresun, Türkiye; b Department of Family Medicine, Faculty of Medicine, Sakarya University, Sakarya, Türkiye.

**Keywords:** burnout, burnout professional, dentists, physicians

## Abstract

This study aimed to determine the burnout levels and identify associated demographic, occupational, and organizational factors among physicians and dentists in Türkiye. Burnout, characterized by emotional exhaustion (EE), depersonalization (DP), and reduced personal accomplishment (PA), is an increasingly critical issue affecting both healthcare providers’ well-being and the quality of healthcare services. A nationwide, cross-sectional, online self-reported survey was conducted between February and August 2024 among 1312 physicians and dentists working in public and private sectors across Türkiye. Data were collected using a structured questionnaire including sociodemographic and professional characteristics, and the Turkish-adapted Maslach Burnout Inventory. Maslach Burnout Inventory subscales scores for EE, DP, and PA were analyzed. Associations between burnout levels and independent variables were evaluated using nonparametric tests, and Bonferroni-adjusted post hoc comparisons. Additionally, hierarchical multivariable linear regression analyses were performed to identify independent predictors of EE, DP, and PA and to adjust for potential confounding factors. Overall, 46.3% of participants had high EE, 16.2% had high DP, and 8.7% had low PA. Higher EE and DP were significantly associated with younger age, fewer years in practice, being unmarried, childless, working in the public sector, lacking administrative experience, exposure to violence or mobbing, involvement in lawsuits, high patient volume, and night shifts. Poor physical work environment, insufficient nonphysician support, and low perceived income were significantly correlated with all 3 subscales, indicating greater burnout risk. Physicians and dentists reporting the intention to leave the profession or to work abroad also exhibited higher burnout scores. In multivariable hierarchical linear regression analyses, intention to leave the profession, exposure to workplace violence, and career-related intentions remained among the strongest independent predictors of EE and DP, while PA showed comparatively weaker associations with sociodemographic and occupational factors. Burnout is highly prevalent among physicians and dentists in Türkiye and is influenced by multiple personal, occupational, and systemic factors. Addressing modifiable contributors (such as workload, workplace safety, staffing adequacy, and income satisfaction) should be prioritized to protect the well-being of healthcare professionals and ensure sustainable, high-quality healthcare delivery.

## 1. Introduction

Burnout was first used in the 1970s in the United States to describe the psychological distress experienced by individuals working in customer service roles. The concept was formally introduced by Freudenberger in 1974, who observed symptoms such as fatigue, frustration, and withdrawal among volunteer healthcare workers. Freudenberger defined burnout as a state of physical and emotional depletion resulting from excessive demands, failure, and exhaustion of internal resources.^[1]^ Today, the most widely recognized and utilized conceptualization of burnout is based on the Maslach Burnout Inventory (MBI), developed by Christina Maslach. Maslach characterized burnout as a syndrome occurring in professions that require intensive interpersonal interaction and defined it across 3 dimensions: emotional exhaustion (EE), depersonalization (DP), and reduced personal accomplishment (PA).^[2]^

Physicians are at particularly high risk of burnout due to their intense professional responsibilities, stressful working conditions, and continuous exposure to emotionally taxing situations.^[3]^ The contributing factors to physician burnout are often excessive workload, long working hours, administrative responsibilities, limited professional autonomy, inadequate organizational support, and exposure to workplace violence or mobbing.^[4]^ Burnout not only affects the individual healthcare provider but also compromises the physician–patient relationship, thereby diminishing the quality of care and disrupting the overall functioning of the healthcare system.^[5–7]^

International studies conducted after the emergence of the COVID-19 pandemic highlight the increase in burnout and related psychological symptoms among healthcare workers during and in the post-pandemic period, demonstrating the lasting workforce pressure and systemic impacts of the pandemic on mental health and working conditions. Studies using large cross-sectional and systematic review designs support these trends.^[8,9]^

In Türkiye, Ergin study revealed that physicians scored higher in EE and DP than individuals in other professions.^[10]^ Despite the growing recognition of these issues, comprehensive national data on the prevalence of burnout among Turkish physicians and dentists, as well as its determinants, remain limited.

The primary objective of this study was to determine the prevalence of burnout syndrome and its subscales (EE, DP, and personal accomplishment) among physicians and dentists working in Türkiye. The secondary objective is to examine the personal, professional, and systemic factors associated with burnout levels in this population.

## 2. Methods

### 2.1. Study design and participants

This cross-sectional descriptive study was part of a specialty thesis project and was conducted between February and August 2024 among physicians and dentists working across various healthcare institutions in Türkiye. To ensure comprehensive representation, we included physicians from both the public and private sectors. A total of 1312 physicians and dentists (1205 physicians and 107 dentists) participated voluntarily in the study. An online self-reported questionnaire was developed using Google Forms and distributed through social media platforms and online professional communication networks frequently used by physicians and dentists in Türkiye. Participation was anonymous and voluntary, with informed consent obtained electronically from all respondents prior to questionnaire completion. The inclusion criteria for this study were physicians and dentists actively working in Türkiye, who agreed to participate in the study.

### 2.2. Data collection tool

The questionnaire consisted of 3 sections:

#### 2.2.1. Sociodemographic information

This section included questions regarding participants’ age, gender, marital status, and whether they had children.

#### 2.2.2. Work-related characteristics

Participants were asked about various aspects of their professional life, including:

- Years of medical practice.
- Professional title (physician or dentist).
- Specialization status of physicians and dentists: specialist/resident or general practitioner.
- Specialty fields of physician specialists/residents: internal medicine sciences, surgical sciences, or basic medical sciences.
- Specialization status of dentists: specialist/resident or general dentist.
- Employment sector: public or private.
- Intention to transition from public to private sector.
- Intention to transition from private to public sector.
- Consideration of leaving the profession.
- History of administrative duty.
- History of being subjected to litigation related to professional practice.
- Exposure to verbal or physical violence during medical practice.
- Exposure to mobbing.
- Physicians’ outpatient clinic work status.
- Average daily number of patients seen by physicians in clinic.
- On-call duty status of physicians.
- Most frequent type of on-call duty assigned to physicians.
- Evaluation of the physical working environment.
- Working with an adequate number of nonphysician healthcare staff.
- Perceived monthly income level.
- Desire to work abroad due to professional reasons.
- Having to give up a hobby due to workload.

#### 2.2.3. Maslach Burnout Inventory

The MBI, originally developed by Maslach and Jackson in 1981, was used in this study to measure burnout levels among physicians. The MBI consists of 22 items divided into 3 subscales: EE, DP, and PA. The EE subscale includes 9 items, the DP subscale consists of 5 items, and the PA subscale contains 8 items. A 7-point likert scale was employed, with responses ranging from “never” (0) to “always” (6). Scoring is done separately for each subscale, with no overall score provided. Higher scores in the EE and DP subscales and lower scores in the PA subscale indicate higher levels of burnout.^[2]^

The MBI was adapted into Turkish by Ergin in 1992. Due to cultural differences, the original 7-point response format was modified into a 5-point Likert scale: 0 = never, 1 = very rarely, 2 = sometimes, 3 = often, 4 = always. The subscale score ranges are as follows: EE (0–36), DP (0–20), and PA (0–32). According to Ergin classification, EE: 0 to 16 (low), 17 to 26 (moderate), ≥27 (high); DP: 0 to 6 (low), 7 to 12 (moderate), ≥13 (high); PA: 0 to 21 (low), 22–28 (moderate), ≥29 (high).^[11]^ In our study, the subscales were calculated as the sum of the relevant items and categorized according to Ergin cutoff values.

### 2.3. Statistical analysis

The study data were analyzed using SPSS for Windows version 22.0 (SPSS Inc., Chicago). Descriptive statistics were presented as mean ± standard deviation (minimum–maximum), and percentages, as appropriate. Categorical variables were compared using the Pearson Chi-square test or Fisher exact test, where applicable. The normality of continuous variables was assessed using both visual methods (histograms and probability plots) and analytical tests (Kolmogorov–Smirnov test and Shapiro–Wilk test). For variables that did not conform to a normal distribution, comparisons between 2 independent groups were performed using the Mann–Whitney *U* test, while comparisons among 3 independent groups were conducted using the Kruskal–Wallis test. When a statistically significant difference was detected, post hoc pairwise comparisons were performed with Bonferroni correction to identify the source of the difference. A *P*-value <.05 was considered statistically significant.

To identify independent predictors of Maslach Burnout Inventory Subscales, hierarchical multivariable linear regression analyses were performed separately for EE, DP, and PA scores. Variables were entered into the models in 4 blocks: sociodemographic variables (gender, marital status, having children); professional characteristics (profession, years of medical practice, employment sector, considering leaving the profession, and administrative role experience); working conditions (work status of physicians in outpatient clinics, on-call duty status of physicians, evaluation of the physical working environment, working with an adequate number of nonphysician healthcare staff, and perception of monthly income); and work-related stressors and career intentions (history of legal proceedings related to profession, exposure to verbal or physical violence in the workplace, exposure to mobbing, desire to work abroad due to professional reasons, having to give up a hobby due to workload). Standardized beta coefficients were calculated to assess the relative strength of associations. Model fit was evaluated using *R*^2^ and change in *R*^2^ statistics. Multicollinearity was assessed using variance inflation factor values. Given the large sample size, linear regression was considered robust to minor deviations from normality of residuals.

### 2.4. Sample size calculation

As of the study period, a total of 234,047 physicians and dentists were actively practicing in Türkiye.^[12]^ The minimum required sample size was calculated as 455 participants, assuming a 50% response distribution, a 3% margin of error, and an 80% confidence level. Ultimately, 1312 physicians and dentists completed the survey, yielding a 97% confidence level.

### 2.5. Ethics approval

The study was approved by the Non-Interventional Clinical Research Ethics Committee of Sakarya University Faculty of Medicine (Approval number: 330215-12, date: January 30, 2024). This study was conducted as a non-interventional, questionnaire-based survey in accordance with the Declaration of Helsinki and relevant national regulations. All participants provided informed consent before participation.

## 3. Results

**Figure 1.**
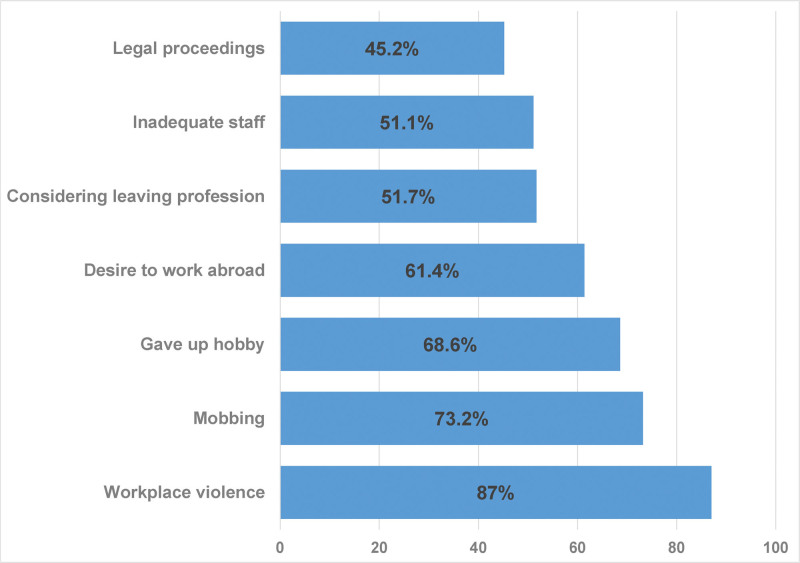
Selected notable findings reported by physicians and dentists. Bars represent the percentage of respondents reporting each condition. Legal proceedings refers to having any ongoing or past legal case due to professional practice. Inadequate staff refers to reporting not working with a sufficient number of nonphysician healthcare personnel. Considering leaving profession refers to contemplating leaving the medical or dental profession. Desire to work abroad refers to wanting to work abroad due to professional adversities. Gave up hobby refers to having to discontinue a regularly practiced hobby because of work-related workload. Mobbing refers to ever having been exposed to workplace bullying by supervisors or colleagues. Workplace violence refers to having been exposed to any verbal or physical violence during the practice of the profession. % = percentage.

A total of 1312 physicians and dentists working in Türkiye participated in the study. The mean age of participants was 37.27 ± 9.35 years, ranging from 23 to 70 years. Of the participants, 30.3% were aged 30 or younger, 38.2% were between 31 to 40 years, and 31.6% were older than 40. The average duration of professional practice was 12.36 ± 9.44 years (range:1–44); 30.4% had more than 15 years of experience, while 31.2% had 5 years or less. Among the participants, 50.5% were female, 70.0% were married, and 55.0% had at least 1 child. Of the participants, 91.8% (n = 1205) were physicians, and 8.2% (n = 107) were dentists. Among physicians, 23.4% (n = 283) were general practitioners, and 76.5% (n = 922) were specialists or residents. Among the latter group, 72.2% (n = 666) were in internal medicine specialties, 23.6% (n = 218) in surgical specialties, and 4.1% (n = 38) in basic medical sciences. Among dentists, 77.6% (n = 83) were general dentists, and 22.4% (n = 24) were dental specialists or residents. A majority (93.1%) of the participants worked in public health institutions. Among 1222 public sector physicians and dentists, 37.1% expressed a desire to transition to the private sector, while 22.2% of 90 private sector physicians and dentists wished to move to the public sector. Overall, 51.7% of all physicians and dentists reported considering leaving the profession. Furthermore, 17.9% (n = 235) had administrative roles currently or in the recent past. Additionally, 45.2% of physicians had been involved in legal proceedings due to their practice, 87.0% had experienced verbal or physical violence, and 73.2% reported being subjected to workplace mobbing by superiors or colleagues (Fig. 1 and Table 1).

**Table 1:** Descriptive characteristics of physicians and dentists.

Variables	Mean ± SD (min–max)/n (%)
Age (yr) (n = 1312)	37.27 ± 9.35 (23–70)
**Age groups (n = 1312**)	
≤30 years	397 (30.3%)
31–40 years	501 (38.2%)
≥41 years	414 (31.6%)
Years of experience as a physicians and dentists (n = 1312)	12.36 ± 9.44 (1–44)
**Years of experience groups (n = 1312**)	
≤5 years	409 (31.2%)
6–15 years	504 (38.4%)
≥16 years	399 (30.4%)
**Gender (n = 1312**)	
Male	649 (49.5%)
Female	663 (50.5%)
**Marital status (n = 1312**)	
Married	918 (70.0%)
Single	394 (30.0%)
Having children (n = 1312)	721 (55.0%)
**Profession (n = 1312**)	
Physician	1205 (91.8%)
Dentist	107 (8.2%)
**Specialization status of physicians (n = 1205**)	
General practitioner	283 (23.4%)
Specialist/resident	922 (76.5%)
**Medical field of physicians (n = 922**)	
Internal medicine sciences	666 (72.2%)
Surgical sciences	218 (23.6%)
Basic medical sciences	38 (4.1%)
**Specialization status of dentists (n = 107**)	
General dentist	83 (77.6%)
Specialist/resident	24 (22.4%)
**Employment sector (n = 1312**)	
Public	1222 (93.1%)
Private	90 (6.9%)
**Desire to switch from public to private sector (n = 1222**)	
Yes	453 (37.1%)
No	769 (62.9%)
**Desire to switch from private to public sector (n = 90**)	
Yes	20 (22.2%)
No	70 (77.8%)
**Considering leaving the profession (n = 1312**)	
Yes	678 (51.7%)
No	634 (48.3%)
**Administrative role experience (n = 1312**)	
Has/had	235 (17.9%)
Never had	1077 (82.1%)
**History of legal proceedings due to medical practice (n = 1312**)	
Yes	593 (45.2%)
No	719 (54.8%)
**Exposure to verbal or physical violence in the workplace (n = 1312**)	
Yes	1142 (87.0%)
No	170 (13.0%)
**Exposure to mobbing (n = 1312**)	
Yes	960 (73.2%)
No	352 (26.8%)

Continuous variables are presented as “mean ± standard deviation (minimum–maximum),” and categorical variables as “n (%).” Totals may not equal 100% due to rounding.

n = number of participants.

Among physicians, 80.1% were working in outpatient clinics. Of 965 physicians working in outpatient settings, 23.5% saw more than 80 patients per day, while 27% saw 40 or fewer. Additionally, 52.5% reported being on call at least once per month. The most common type of on-call duty was departmental (50.7%), followed by emergency (25.0%), on-call/other duties (24.3%). Regarding the physical work environment, 56.9% described it as poor, and only 17.5% considered it good. About 51.1% reported a lack of adequate nonphysician healthcare staff. While 24.8% perceived their income as low, only 9.4% rated it as good. A total of 61.4% expressed a desire to work abroad for professional reasons, and 68.6% had to give up a regular hobby due to work-related obligations (Fig. 1 and Table 2).

**Table 2:** Some characteristics related to physicians’ and dentists’ working conditions.

Variables	n (%)
**Work status of physicians in outpatient clinics (n = 1205**)	
Working	965 (80.1%)
Not working	240 (19.9%)
**Average daily number of patients per physician in outpatient clinic (n = 965**)	
0–40 patients	267 (27.6%)
41–80 patients	472 (48.9%)
>80 patients	226 (23.4%)
**On-call duty status of physicians (n = 1205**)	
On call	633 (52.5%)
Not on call	572 (47.5%)
**Most frequent type of on-call duty assigned to physicians (n = 633**)	
Departmental duty	321 (50.7%)
Emergency department duty	158 (25.0%)
On-call/other duties	154 (24.3%)
**Evaluation of the physical working environment (n = 1312**)	
Good	229 (17.5%)
Moderate	337 (25.7%)
Poor	746 (56.9%)
**Working with an adequate number of nonphysician or non-dentist healthcare staff (n = 1312**)	
Yes	641 (48.9%)
No	671 (51.1%)
**Perception of monthly income (n = 1312**)	
Good	123 (9.4%)
Moderate	864 (65.9%)
Poor	325 (24.8%)
**Desire to work abroad due to professional reasons (n = 1312**)	
Yes	806 (61.4%)
No	506 (38.6%)
**Having to give up a hobby due to workload (n = 1312**)	
Yes	900 (68.6%)
No	412 (31.4%)

Continuous variables are presented as “mean ± standard deviation (minimum–maximum),” and categorical variables as “n (%).” Totals may not equal 100% due to rounding.

n = number of participants.

The mean MBI subscale scores were 22.28 ± 8.29 for emotional exhaustion (EE), 8.68 ± 4.75 for DP, and 18.87 ± 5.95 for personal accomplishment (PA) (Table 3). Among the participants, 46.3% had high EE, 43.2% moderate, and 10.5% low. For DP, 16.2% had high scores, 49.0% moderate, and 34.8% low. In the PA dimension, 33.9% had high scores, 57.4% moderate, and 8.7% low (Table 4).

**Table 3:** Maslach Burnout Inventory subscales scores mean and burnout level of the physicians and dentists.

Subscale (n = 1312)	Mean ± SD (min–max)	Burnout level (score range)
Emotional exhaustion	22.28 ± 8.29 (0–36)	Moderate burnout (17–26)
Depersonalization	8.68 ± 4.75 (0–20)	Moderate burnout (7–12)
Personal accomplishment	18.87 ± 5.95 (0–32)	Low burnout (0–21)

n = number of participants; SD = standard deviation.

**Table 4:** Physicians’ and dentists’ burnout levels according to the Maslach Burnout Inventory.

MBI subscale	Burnout level (score range)	n (%)
Emotional exhaustion	Low (0–11)	138 (10.5%)
	Moderate (12–23)	567 (43.2%)
	High (24–36)	607 (46.3%)
Depersonalization	Low (0–6)	456 (34.8%)
	Moderate (7–13)	643 (49.0%)
	High (14–20)	213 (16.2%)
Personal accomplishment	Low (0–10)	114 (8.7%)
	Moderate (11–21)	753 (57.4%)
	High (22–32)	445 (33.9%)

MBI = Maslach Burnout Inventory; n = number of participants.

Statistically significant differences were found in EE and DP scores across age groups (EE: 22.92 ± 8.43 vs 20.93 ± 8.05, mean difference (MD) = 1.99, *P* < .001; DP: 9.56 ± 4.82 vs 7.48 ± 4.52, MD = 2.08, *P* < .001), with physicians and dentists over 40 years having significantly lower scores than younger age groups. However, PA scores did not differ significantly by age groups (*P* = .68). Similar patterns were observed regarding years in practice: those with >15 years of experience had significantly lower EE and DP scores compared to those with 0 to 15 years (EE: 22.67 ± 7.86 vs 21.01 ± 8.89, MD = 1.66, *P* = .003; DP: 9.46 ± 4.39 vs 7.62 ± 5.01, MD = 1.84, *P* < .001). No significant difference was found in PA scores (*P* = .89). Female physicians and dentists had significantly higher EE scores than males (EE: 23.02 ± 8.41 vs 21.52 ± 8.11, MD = 1.50, *P* = .002), while no gender differences were observed in DP and PA (DP: *P* = .43, PA: *P* = .33). Unmarried physicians and dentists had significantly higher EE and DP scores than married ones (EE: 23.17 ± 7.93 vs 21.89 ± 8.41, MD = 1.28, *P* = .02; DP: 9.26 ± 4.60 vs 8.42 ± 4.79, MD = 0.84, *P* < .001), but PA scores did not differ (*P* = .47). Physicians and dentists with children had lower EE and DP scores than those without (EE: 23.21 ± 7.81 vs 21.51 ± 8.59, MD = 1.70, *P* < .001; DP: 9.30 ± 4.54 vs 8.16 ± 4.86, MD = 1.14, *P* < .001), with no significant differences in PA (*P* = .05). Physicians had lower EE and DP scores than dentists (EE: 22.09 ± 8.22 vs 24.35 ± 8.80, MD = 2.26, *P* = .01; DP: 8.50 ± 4.68 vs 10.67 ± 5.06, MD = 2.17, *P* < .001), but PA scores did not differ (*P* = .05). Among physicians, specialists had lower EE than nonspecialists (EE: 21.63 ± 8.14 vs 23.60 ± 8.29, MD = 1.97, *P* < .001), with no differences in DP and PA (DP: *P* = .11; PA: *P* = .46). Among specialist/resident physicians, those working in internal medicine sciences had significantly higher EE scores than those in surgical sciences (EE: 22.08 ± 8.04 vs 20.54 ± 7.28, MD = 1.54, *P* = .02), while DP and PA scores were not significantly different (DP: *P* = .09; PA: *P* = .36). Among dentists, specialists had lower DP scores than general dentists (DP: 9.00 ± 4.76 vs 11.16 ± 5.07, MD = 2.16, *P* = .04); no differences were found in EE and PA (EE: *P* = .08; PA: *P* = .08) (Table 5).

**Table 5:** Distribution of Maslach Burnout Inventory scores according to descriptive characteristics of physicians and dentists.

Variables	Maslach Burnout Inventory mean ± SD (min–max)		
	Emotional exhaustion	Depersonalization	Personal accomplishment
**Age group**			
≤30 years (n = 397)	22.92 ± 7.90 (0–36)**	9.56 ± 4.44 (0–20)**	18.98 ± 5.16 (1–31)
31–40 years (n = 501)	22.88 ± 8.1 (0–36)**	8.96 ± 4.64 (0–20)**	18.91 ± 5.94 (0–32)
≥41 years (n = 414)	20.93 ± 8.82 (0–36)††	7.48 ± 4.94 (0–20)††	18.71 ± 6.64 (0–32)
*P* †	<.001*	<.001*	.68
**Years of medical practice group**			
≤5 years (n = 409)	22.67 ± 7.86 (0–36)**	9.46 ± 4.39 (0–20)**	18.92 ± 5.11 (4–31)
6–15 years (n = 504)	22.97 ± 8.04 (0–36)**	8.88 ± 4.68 (0–20)**	18.88 ± 5.98 (0–32)
≥16 years (n = 399)	21.01 ± 8.89 (0–36)††	7.62 ± 5.01 (0–20)††	18.79 ± 6.69 (0–32)
*P* †	.003*	<.001*	.89
**Gender**			
Male (n = 649)	21.52 ± 8.57 (0–36)	8.79 ± 4.90 (0–20)	18.68 ± 6.18 (0–32)
Female (n = 663)	23.02 ± 7.94 (0–36)	8.56 ± 4.59 (0–20)	19.05 ± 5.72 (1–32)
*P* ‡	.002*	.43	.33
**Marital status**			
Married (n = 918)	21.89 ± 8.41 (0–36)	8.42 ± 4.79 (0–20)	18.94 ± 6.06 (0–32)
Unmarried (n = 394)	23.17 ± 7.93 (0–36)	9.26 ± 4.60 (0–20)	18.69 ± 5.68 (1–32)
*P* ‡	0.02*	<.001*	.47
**Having children**			
Yes (n = 721)	21.51 ± 8,59 (0–36)	8,16 ± 4,86 (0–20)	19,08 ± 6,35 (0–32)
No (n = 591)	23.21 ± 7,81 (0–36)	9,30 ± 4,54 (0–20)	18,60 ± 5,42 (0–32)
*P* ‡	<.001*	<.001*	.05
**Profession**			
Physician (n = 1205)	22.09 ± 8.22 (0–36)	8.50 ± 4.68 (0–20)	18.95 ± 5.99 (0–32)
Dentist (n = 107)	24.35 ± 8.82 (0–36)	10.67 ± 5.06 (0–20)	17.98 ± 5.41 (7–31)
*P* ‡	.01*	<.001*	.05
**Specialization status of physicians**			
General practitioner (n = 283)	23.60 ± 8.29 (1–36)	8.86 ± 4.72 (0–20)	18.85 ± 5.89 (4–32)
Specialist/resident (n = 922)	21.63 ± 8.14 (0–36)	8.39 ± 4.66 (0–20)	18.98 ± 6.03 (0–32)
*P* ‡	<.001*	.11	.46
**Medical field of physicians**			
Internal medicine sciences (n = 666)	22.08 ± 8.04 (0–36)**	8.58 ± 4.62 (0–20)	19.00 ± 5.99 (0–32)
Surgical sciences (n = 218)	20.54 ± 8.22 (0–36)††	7.94 ± 4.78 (0–20)	19.18 ± 6.08 (3–32)
Basic medical sciences (n = 38)	19.92 ± 8.98 (0–35)**^,^††	7.68 ± 4.68 (0–20)	17.39 ± 6.28 (0–27)
*P* †	.02*	.09	.36
**Specialization status of dentists**			
General dentist (n = 83)	25.17 ± 8.59 (3–36)	11.16 ± 5.07 (0–20)	17.46 ± 5.10 (8–31)
Specialist/resident (n = 24)	21.50 ± 9.18 (5–36)	9.00 ± 4.76 (2–20)	19.79 ± 6.13 (7–30)
*P* ‡	.08	.04*	.08

Variables are presented as “mean ± standard deviation (minimum–maximum).”

n = number of participants; SD = standard deviation.

† Kruskal–Wallis test.

‡ Mann–Whitney *U* test.

* *P* < .05. Different symbols (

** ,

†† ) indicate statistically significant differences between groups based on post hoc tests (*P* < .05). Values sharing the same symbols are not significantly different from each other.

**Figure 2.**
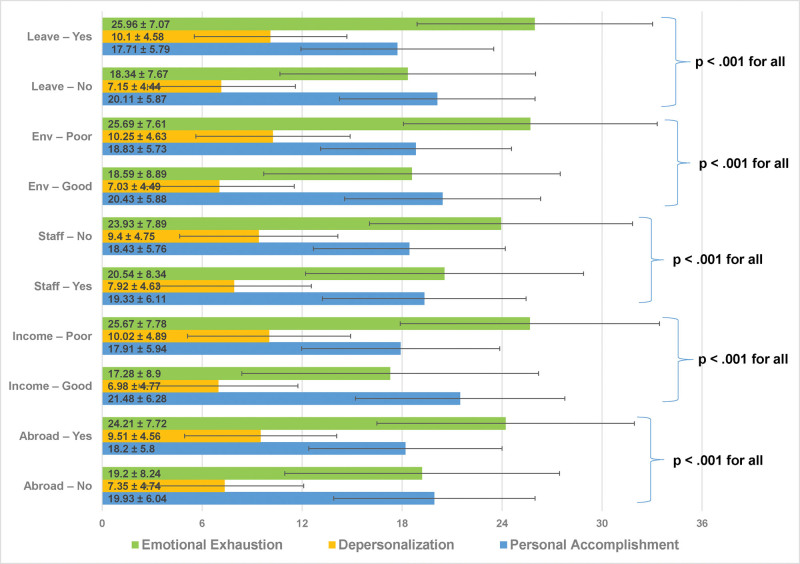
Variables significantly associated with all 3 Maslach Burnout Inventory subscales. Bar charts illustrating variables that were statistically significantly associated (*P* < .05) with all 3 Maslach Burnout Inventory subscales. Bars represent mean values, and error bars indicate standard deviation. Leave: yes/no refers to contemplating leaving the medical or dental profession. Env: poor/good refers to refers to physicians’ or dentists’ perception of the physical conditions of their work environment as poor or good. Staff: no/yes refers to reporting working or not working with a sufficient number of nonphysician healthcare personnel. Income: poor/good refers to perceived adequacy of income. Abroad: yes/no refers to desire to work or not work abroad due to professional adversities. Green bars represent emotional exhaustion, orange bars represent depersonalization, and blue bars represent personal accomplishment. All comparisons between groups were statistically significant (*P* < .001 for all subscales).

Public-sector physicians and dentists had significantly higher EE and DP scores than those in the private sector (EE: 22.61 ± 8.14 vs 17.73 ± 8.98, MD = 4.88, *P* < .001; DP: 8.82 ± 4.73 vs 6.73 ± 4.63, MD = 2.09, *P* < .001), with no differences in PA (*P* = .13). Among public physicians and dentists, those desiring to move to the private sector had higher EE and DP scores than those who did not (EE: 22.61 ± 8.14 vs 17.73 ± 8.98, MD = 4.88, *P* < .001; DP: 8.82 ± 4.73 vs 6.73 ± 4.63, MD = 2.09, *P* < .001). Among private-sector physicians and dentists, those desiring to return to the public sector had significantly higher PA scores (PA: 22.25 ± 5.31 vs 19.07 ± 6.64, MD = 3.18, *P* = .04), though EE and DP scores did not differ (EE: *P* = .94; DP: *P* = .81). Physicians and dentists considering leaving the profession had higher EE and DP scores and lower PA scores (EE: 25.96 ± 7.87 vs 18.34 ± 7.42, MD = 7.62, *P* < .001; DP: 10.10 ± 4.55 vs 7.15 ± 4.18, MD = 2.95, *P* < .001; PA: 17.71 ± 5.60 vs 20.11 ± 5.83, MD = 2.40, *P* < .001). Those who had never held an administrative position had higher EE and DP scores than those with such experience (EE: 22.56 ± 8.26 vs 21.00 ± 8.30, MD = 1.56, *P* = .01; DP: 8.90 ± 4.72 vs 7.60 ± 4.74, MD = 1.30, *P* < .001), but PA scores did not differ (*P* = .43). Physicians and dentists involved in legal proceedings had higher EE and DP scores (EE: 22.92 ± 8.21 vs 21.74 ± 8.32, MD = 1.18, *P* = .01; DP: 9.11 ± 4.81 vs 8.32 ± 4.67, MD = 0.79, *P* = .01), with no difference in PA (*P* = .92). Those exposed to violence had significantly higher EE and DP scores (EE: 23.11 ± 7.92 vs 16.65 ± 8.56, MD = 6.46, *P* < .001; DP: 9.17 ± 4.62 vs 5.38 ± 4.23, MD = 3.79, *P* < .001), with no difference in PA (*P* = .79). Those exposed to mobbing had significantly higher EE and DP scores (EE: 23.63 ± 7.78 vs 18.57 ± 8.51, MD = 5.06, *P* < .001; DP: 9.13 ± 4.69 vs 7.45 ± 4.70, MD = 1.68, *P* < .001), with no difference in PA (*P* = .06) (Fig. 2 and Table 6).

**Table 6:** Distribution of Maslach Burnout Inventory scores according to occupational characteristics of physicians and dentists.

Variables	Maslach Burnout Inventory Mean ± SD (min–max)		
	Emotional exhaustion	Depersonalization	Personal accomplishment
**Employment sector**			
Public (n = 1222)	22.61 ± 8.14 (0–36)	8.82 ± 4.73 (0–20)	18.80 ± 5.91 (0–32)
Private (n = 90)	17.73 ± 8.98 (0–36)	6.73 ± 4.63 (0–20)	19.78 ± 6.48 (4–32)
*P* †	<.001*	<.001*	.13
**Desire to switch from public to private sector**			
Yes (n = 453)	24.33 ± 7.38 (0–36)	9.70 ± 4.62 (0–20)	18.46 ± 5.61 (0–32)
No (n = 769)	21.60 ± 8.40 (0–36)	8.30 ± 4.71 (0–20)	19.00 ± 6.07 (0–32)
*P* †	<.001*	<.001*	.14
**Desire to switch from private to public sector**			
Yes (n = 20)	17.75 ± 8.82 (1–36)	6.85 ± 4.38 (1–19)	22.25 ± 5.31 (13–31)
No (n = 70)	17.73 ± 9.09 (0–36)	6.70 ± 4.73 (0–20)	19.07 ± 6.64 (4–32)
*P* †	.94	.81	.04*
**Considering leaving the profession**			
Yes (n = 678)	25.96 ± 7.07 (0–36)	10.10 ± 4.58 (0–20)	17.71 ± 5.79 (0–32)
No (n = 634)	18.34 ± 7.67 (0–36)	7.15 ± 4.44 (0–20)	20.11 ± 5.87 (0–32)
*P* †	<.001*	<.001*	<.001*
**Administrative role experience**			
Has/had administrative roles (n = 235)	21.00 ± 8.30 (0–36)	7.66 ± 4.74 (0–20)	19.17 ± 6.43 (0–32)
Never had administrative roles (n = 1077)	22.56 ± 8.26 (0–36)	8.90 ± 4.72 (0–20)	18.80 ± 5.84 (0–32)
*P* †	.01*	<.001*	.43
**History of legal proceedings related to profession**			
Yes (n = 593)	22.92 ± 8.21 (0–36)	9.11 ± 4.81 (0–20)	18.89 ± 6.12 (0–32)
No (n = 719)	21.74 ± 8.32 (0–36)	8.32 ± 4.67 (0–20)	18.84 ± 5.81 (0–32)
*P* †	.01*	.01*	.92
**Exposure to verbal or physical violence in the workplace**			
Yes (n = 1142)	23.11 ± 7.92 (0–36)	9.17 ± 4.62 (0–20)	18.88 ± 5.79 (3–32)
No (n = 170)	16.65 ± 8,56 (0–36)	5.38 ± 4.23 (0–20)	18.81 ± 6.94 (0–32)
*P* †	<.001*	<.001*	.79
**Exposure to mobbing**			
Yes (n = 960)	23.63 ± 7.78 (0–36)	9.13 ± 4.69 (0–20)	18.67 ± 5.96 (0–32)
No (n = 352)	18.57 ± 8.51 (0–36)	7.45 ± 4.70 (0–20)	19.40 ± 5.90 (0–32)
*P* †	<.001*	<.001*	.06

Variables are presented as “mean ± standard deviation (minimum–maximum)”.

n = number of participants; SD = standard deviation.

† Mann–Whitney *U* test.

* *P* < .05.

Physicians working in outpatient clinics had significantly higher EE and PA scores than those who did not (EE: 22.35 ± 8.20 vs 21.05 ± 8.21, MD = 1.30, *P* = .02; PA: 19.15 ± 6.03 vs 18.11 ± 5.79, MD = 1.04, *P* = .01), with no significant difference in DP (*P* = .28). Among these physicians, EE scores increased progressively with higher patient load, with significant differences observed across all 3 groups (*P* < .001 for all; >80 vs ≤40 patients/day: 24.54 ± 8.12 vs 19.36 ± 7.65, MD = 5.18). For DP, physicians seeing 0 to 40 patients/day had significantly lower scores than those seeing 41 to 80 or >80 patients/day (DP: 7.61 ± 4.70 vs 9.17 ± 4.69, MD = 1.56, *P* < .001), whereas no significant difference was observed between the latter 2 groups. No significant differences were found in PA scores (*P* = .12). Physicians with on-call duty had higher EE and DP and lower PA scores (EE: 23.41 ± 7.87 vs 20.64 ± 8.36, MD = 2.77, *P* < .001; DP: 9.29 ± 4.58 vs 7.62 ± 4.64, MD = 1.67, *P* < .001; PA: 18.55 ± 5.74 vs 19.38 ± 6.23, MD = 0.83, *P* = .01). Emergency duty was associated with higher EE and DP compared to other on-call duty types (EE: 25.13 ± 7.64 vs 22.78 ± 7.67, MD = 2.35, *P* = .01; DP: 10.28 ± 4.55 vs 8.94 ± 4.47, MD = 1.34, *P* = .01), with no significant difference in PA (*P* = .21). All 3 MBI subscales differed significantly according to perceived quality of the physical working environment (EE: 25.69 ± 7.61 vs 18.59 ± 8.39, MD = 7.10, *P* < .001; DP: 10.25 ± 4.63 vs 7.03 ± 4.49, MD = 3.22, *P* < .001; PA: 18.83 ± 5.73 vs 20.43 ± 5.88, MD = 1.60, *P* < .001). EE and DP scores increased progressively from good to poor environments, whereas PA scores were significantly higher among those reporting a good work environment compared with moderate and poor environments, with no difference between the latter 2 groups. Those reporting insufficient nonphysician support had higher EE and DP and lower PA scores (EE: 23.93 ± 7.89 vs 20.54 ± 8.34, MD = 3.39, *P* < .001; DP: 9.40 ± 4.75 vs 7.92 ± 4.63, MD = 1.48, *P* < .001; PA: 18.43 ± 5.76 vs 19.33 ± 6.11, MD = 0.90, *P* = .003). Perceived monthly income was significantly associated with all 3 MBI subscales (good vs poor: EE 17.28 ± 8.00 vs 25.67 ± 7.78, MD = 8.39; DP 6.98 ± 4.77 vs 10.03 ± 4.89, MD = 3.05; PA 21.48 ± 6.28 vs 17.91 ± 5.94, MD = 3.57; *P* < .001 for all). EE and DP scores increased progressively as income perception worsened, whereas PA scores decreased across the same gradient, with all pairwise comparisons showing significant differences. Physicians who wished to work abroad had higher EE and DP scores and lower PA scores (EE: 24.21 ± 7.72 vs 19.20 ± 8.24, MD = 5.01, *P* < .001; DP: 9.51 ± 4.56 vs 7.35 ± 4.74, MD = 2.16, *P* < .001; PA: 18.20 ± 5.80 vs 19.93 ± 6.04, MD = 1.73, *P* < .001). Similarly, those who reported giving up a hobby due to workload also had higher EE and DP scores (EE: 23.84 ± 7.74 vs 18.86 ± 8.43, MD = 4.98, *P* < .001; DP: 9.17 ± 4.69 vs 7.61 ± 4.70, MD = 1.56, *P* < .001), with no significant difference in PA (*P* = .06) (Fig. 2 and Table 7).

**Table 7:** Maslach Burnout Inventory scores by physicians’ and dentists’ work conditions and professional factors.

Variables	Maslach Burnout Inventory Mean ± SD (min–max)		
	Emotional exhaustion	Depersonalization	Personal accomplishment
**Work status of physicians in outpatient clinics**			
Yes (n = 965)	22.35 ± 8.20 (0–36)	8.58 ± 4.69 (0–20)	19.15 ± 6.03 (0–32)
No (n = 240)	21.05 ± 8.21 (0–36)	8.17 ± 4.66 (0–20)	18.11 ± 5.79 (0–32)
*P* ‡	.02*	.28	.01*
**Average daily number of patients per physician in outpatient clinic**			
0–40 (n = 267)	19.36 ± 8.26 (0–36)**	7.44 ± 4.59 (0–20)**	19.74 ± 5.91 (0–32)
41–80 (n = 472)	23.00 ± 7.76 (3–36)††	8.83 ± 4.51 (0–20)††	19.01 ± 6.02 (3–32)
>80 (n = 226)	24.54 ± 8.09 (0–36)‡‡	9.41 ± 4.92 (0–20)††	18.77 ± 6.14 (0–32)
*P* †	<.001*	<.001*	.12
**On-call duty status of physicians**			
Yes (n = 633)	23.41 ± 7.87 (0–36)	9.29 ± 4.58 (0–20)	18.55 ± 5.74 (0–32)
No (n = 572)	20.64 ± 8.36 (0–36)	7.62 ± 4.64 (0–20)	19.38 ± 6.23 (0–32)
*P* ‡	<.001*	<.001*	.01*
**Most frequent type of on-call duty assigned to physicians**			
Departmental duty (n = 321)	22.78 ± 7.67 (1–36)**	8.94 ± 4.47 (0–20)**	18.74 ± 5.68 (0–32)
Emergency duty (n = 158)	25.13 ± 7.64 (6–36)††	10.28 ± 4.55 (0–20)††	18.64 ± 5.60 (4–32)
Standby duty/another duty (n = 154)	22.95 ± 8.29 (0–36)**	9.01 ± 4.69 (0–20)**	18.08 ± 6.04 (0–32)
*P* †	.01*	.01*	.21
**Evaluation of the physical working environment**			
Good (n = 229)	18.59 ± 8.89 (0–36)**	7.03 ± 4.49 (0–20)**	20.43 ± 6.58 (0–32)**
Moderate (n = 746)	21.86 ± 7.81 (0–36)††	8.47 ± 4.67 (0–20)††	17.89 ± 5.78 (3–32)††
Poor (n = 337)	25.69 ± 7.61 (2–36)‡‡	10.25 ± 4.63 (0–20)‡‡	18.83 ± 5.73 (1–32)‡‡
*P* †	<.001*	<.001*	<.001*
**Working with an adequate number of nonphysician healthcare staff**			
Yes (n = 641)	20.54 ± 8.34 (0–36)	7.92 ± 4.63 (0–20)	19.33 ± 6.11 (0–32)
No (n = 671)	23.93 ± 7.89 (0–36)	9.40 ± 475 (0–20)	1843 ± 5.76 (0–32)
*P* ‡	<.001*	<.001*	.003*
**Perception of monthly income**			
Good (n = 123)	17.28 ± 8.90 (0–36)**	6.98 ± 4.77 (0–20)**	21.49 ± 6.28 (0–32)**
Moderate (n = 864)	21.71 ± 7.89 (0–36)††	8.41 ± 4.57 (0–20)††	18.85 ± 5.80 (0–32)††
Poor (n = 325)	25.67 ± 7.78 (0–36)‡‡	10.02 ± 4.89 (0–20)‡‡	17.91 ± 5.94 (0–32)‡‡
*P* †	<.001*	<.001*	<.001*
**Desire to work abroad due to professional reasons**			
Yes (n = 806)	24.21 ± 7.72 (1–36)	9.51 ± 4.56 (0–20)	18.20 ± 5.80 (0–32)
No (n = 506)	19.20 ± 8.24 (0–36)	7.35 ± 4.74 (0–20)	19.93 ± 6.04 (0–32)
*P* ‡	<.001*	<.001*	<.001*
**Having to give up a hobby due to workload**			
Yes (n = 900)	23.84 ± 7.74 (0–36)	9.17 ± 4.69 (0–20)	18.64 ± 5.85 (0–32)
No (n = 412)	18.86 ± 8.43 (0–36)	7.61 ± 4.70 (0–20)	19.35 ± 6.14 (0–32)
*P* ‡	<.001*	<.001*	.06

Variables are presented as “mean ± standard deviation (minimum–maximum).”

n = number of participants; SD = standard deviation.

† Kruskal–Wallis test.

‡ Mann–Whitney *U* test.

* *P* < .05. Different symbols (

** ,

†† ,

‡‡ ) indicate statistically significant differences between groups based on post hoc tests (*P* < .05). Values sharing the same symbols are not significantly different from each other.

Hierarchical multivariable linear regression analyses were performed separately for EE, DP, and PA (Table 8). Results are presented as standardized β coefficients and corresponding *P* values. In the final model, the included variables explained 35.9% of the variance in EE (*R*^2^ = 0.359). After full adjustment, EE was independently associated with gender (β = 0.096, *P* < .001), employment sector (β = 0.082, *P* < .001), considering leaving the profession (β = 0.324, *P* < .001), on-call duty status (β = 0.059, *P* = .014), working with an adequate number of nonphysician healthcare staff (β = ‐0.068, *P* = .004), perception of monthly income (β = 0.079, *P* = .001), exposure to verbal or physical violence in the workplace (β = 0.105, *P* < .001), exposure to mobbing (β = 0.108, *P* < .001), desire to work abroad due to professional reasons (β = 0.090, *P* < .001), and having to give up hobbies due to workload (β = 0.104, *P* < .001). For DP, the final hierarchical model accounted for 23.6% of the variance (*R*^2^ = 0.236). Independent predictors included profession (physician or dentist) (β = 0.097, *P* < .001), years of medical practice (β = −0.164, *P* < .001), considering leaving the profession (β = 0.211, *P* < .001), on-call duty status (β = 0.073, *P* = .006), evaluation of the physical working environment (β = 0.084, *P* = .002), exposure to verbal or physical violence in the workplace (β = 0.177, *P* < .001), and desire to work abroad due to professional reasons (β = 0.075, *P* = .005). The explanatory power of the model for PA was comparatively limited, with 6.2% of the variance explained (*R*^2^ = 0.062). Independent predictors included considering leaving the profession (β = −0.156, *P* < .001), work status in outpatient clinics (β = 0.073, *P* = .008), evaluation of the physical working environment (β = −0.070, *P* = .019), perception of monthly income (β = −0.065, *P* = .029), exposure to verbal or physical violence in the workplace (β = 0.060, *P* = .041), and desire to work abroad due to professional reasons (β = −0.063, *P* = .034).

**Table 8:** Hierarchical multivariable linear regression analyses for Maslach Burnout Inventory subscales.

Variables	Emotional exhaustion	Depersonalization	Personal accomplishment
Gender	0.096 (0.829 to 2.338), *P* < .001		
Profession (physician or dentist)		0.097 (0.827 to 2.526), *P* < .001	
Years of medical practice		‐0.164 (‐1.389 to ‐0.596), *P* < .001	
Employment sector	0.082 (1.181 to 4.191), *P* < .001		
Considering leaving the profession	0.324 (4.562 to 6.167), *P* < .001	0.211 (1.498 to 2.509), *P* < .001	‐0.156 (‐2.551 to ‐1.157), *P* < .001
Work status of physicians in outpatient clinics			0.073 (0.282 to 1.2925), *P* = .008
On-call duty status of physicians	0.059 (0.200 to 1.767), *P* = .014	0.073 (0.199 to 1.186), *P* = .006	
Evaluation of the physical working environment		0.084 (0.224 to 0.998), *P* = .002	‐0.070 (‐1.176 to ‐0.107), *P* = .019
Working with an adequate number of nonphysician healthcare staff	‐0.068 (‐1.893 to ‐0.364), *P* = .004		
Perception of monthly income	0.079 (0.446 to 1.866), *P* = .001		‐0.065 (‐1.304 to ‐0.071), *P* = .029
Exposure to verbal or physical violence in the workplace	0.105 (1.413 to 3.763), *P* < .001	0.177 (1.764 to 3.244), *P* < .001	0.060 (0.042 to 2.083), *P* = .041
Exposure to mobbing	0.108 (1.142 to 2.902), *P* < .001		
Desire to work abroad due to professional reasons	0.090 (0.715 to 2.341), *P* < .001	0.075 (0.215 to 1.238), *P* = .005	‐0.063 (‐1.472 to ‐0.060), *P* = .034
Having to give up a hobby due to workload	0.104 (1.017 to 2.685), *P* < .001		

Values are presented as standardized β coefficients, 95% confidence intervals for unstandardized regression coefficients (*B*), and *P*-values derived from the final hierarchical models. Model fit (*R*^2^): emotional exhaustion = 0.359, depersonalization = 0.236, personal accomplishment = 0.062.

## 4. Discussion

The physicians and dentists in Turkiye (in this nationwide study) had moderate levels of EE and DP and low levels of personal PA (Table 3). In line with our findings, a study conducted on 406 Turkish physicians indicated moderate EE and DP and low PA.^[13]^ Almost half of our participants were high EE, moderate DP, and PA (Table 4). The same results were found in physicians in Ethiopia and South Korea where over half reported high EE and DP.^[14,15]^ These findings support the fact that the medical profession is exceptionally prone to burnouts.

The item that was highly related to burnout was age, years of practice, marital status, parenthood, dentist, employment sector, administration responsibilities, lawsuits, exposure to violence or mobbing, patient count, and night shifts. Moreover, the desire to leave, shift work, poor working environment, lack of nonphysician support, and perceived low income were linked to all the 3 dimensions of burnout. They are expected to impact all the MBI elements in a way that is most widespread and must be tackled at both the personal and the corporate level. Importantly, these findings are descriptive in nature and reflect associations observed in a cross-sectional design; therefore, causal inferences cannot be established.

In line with the literature night shift physicians especially in emergency departments had elevated EE and DP.^[11,16,17]^ We also found that this group also suffered negatively regarding PA scores. The doctors who felt that their working environment was physically substandard experienced increased burnout in all dimensions. On the same note, poor nonphysician support and low perceived income were also associated with burnout. Though this particular association has not been thoroughly investigated, The Organization for Economic Cooperation and Development (OECD) statistics, featuring that Turkiye has one of the lowest nurse-densities of all OECD countries (2.8 vs 9.2 per 1000 population), and the established impact of financial dissatisfaction corroborate our results.^[18]^ These structural indicators may partially contextualize the elevated burnout levels observed in our cohort, although longitudinal research would be required to clarify causal pathways. Enhancing work conditions, adequate staffing, and income dissatisfaction can thus be critical measures that can help reduce burnout.

There was more burnout in younger physicians, as it has been reported before.^[14,19,20]^ The correlation given between age and professional experience and the burnout is negative, which can be attributed to insufficient coping mechanisms, lack of confidence, and stress of the workload at the start of a career. However, while reduced coping experience and early-career stress may plausibly contribute to this pattern, the present study design does not allow definitive conclusions regarding underlying mechanisms. The inconsistencies in previous Turkish researches could be due to the institutional differences.^[21,22]^ We also find that similar to the results of the female, unmarried, and childless physicians, they report more burnout,^[20–25]^ which may be attributed to the lack of social support and gender pressures.

Mobbing, violence, and litigation were found to be some of the influential factors that contributed to burnout. Consistent with Peruvian results where psychological violence was related to burnout among physicians, our results revealed that burnout was significantly higher among physicians who encountered mobbing or violence.^[26]^ The same findings were presented among the Turkish emergency physicians.^[27]^ Nine out of 10 participants had experienced professional lawsuits, almost half of them had experienced violence, and 7 of 10 mobbing highlighting the severity of such occupational hazards. There was also increased burnout among physicians who were involved in lawsuits, which provides support to prior data by U.S. surgeons.^[28]^ Taken together, these data points to the cruel environment in which the doctors have to operate, and the dire necessity of the institutional changes that will provide the safety and the legal security of the workplace. These findings underscore the importance of enforceable workplace violence prevention policies and institutional legal support mechanisms as part of systemic burnout mitigation strategies.

When it comes to professional specialization, the general practitioners were found to be more affected by burnout compared to specialists.^[29]^ The burnout among internal medicine physicians was higher than that of surgical specialists,^[16]^ which is probably due to the high number of outpatients characteristic of internal medicine. As in our case, an Ethiopian study discovered that the more patients one sees per week, the higher EE and DP.^[14]^ Burnout was much higher in physicians in our cohort with >40 patients per day. Such results are consistent with the initial definition of Maslach and Jackson that defined burnout as the most prevalent in the jobs that demand face-to-face interaction.^[2]^ There was also a higher level of burnout among dentists, who work in continuous contact with patients compared to physicians which is also supported by the previous research.^[30]^ On the other hand, the individuals who had administrative responsibilities showed lower burnout rates, which may have been as a result of less clinical intensity, in line with earlier findings.^[21]^ From a system-level perspective, regulating patient load thresholds and optimizing physician-to-support-staff ratios may represent concrete interventions to alleviate workload-driven burnout.

Differences were also important in terms of sector. There was a higher level of burnout in physicians and dentists in the public sector compared to the private sector. This is in line with the past researches that have indicated that there is higher burnout among the public dentists.^[13,31]^ Public employees that indicated their intentions to switch to the private sector also showed a greater burnout among public workers, indicating that they were not happy with the high workload, bureaucracy, and pressure of patients at the public institutions. This tendency can pose a risk to the sustainability of the state healthcare system and represents the structural problem behind professional burnout.

The other interesting observation was that the doctors who were forced to give up hobbies due to workload exhibited increased burnout. About 70% of the respondents said they had abandoned a hobby because of career reasons. This finding extends beyond a simple lifestyle choice and may serve as a measurable indicator of work–life imbalance and erosion of recovery time. Loss of regular leisure activity reflects reduced psychological detachment from work, which is a key component of resilience and mental well-being. This is a significant discovery that allows illuminating on the loss of personal time and life balance of healthcare workers, which is rarely studied.

Over 50% of the respondents indicated that they planned to quit the profession, which is also similar to the past Turkish statistics.^[22,32]^ Similarly, >50% said they would wish to work in a foreign country probably due to the ambitions of seeking improved working and living standards. This increasing desire to emigrate or switch professions demonstrates an institutional crisis in the healthcare labor force in Türkiye. And with a physician to 1000 population ratio of 2.2 compared to the OECD average of 3.7, overwork and rampant violence (9 of 10 respondents reported this) contribute to this general disenchantment.^[18]^

Altogether, our results indicate that burnout among doctors and dentists in Türkiye does not solely rely on the personal and demographic factors but also on structural and organizational stress factors. Overworking, staff understaffing, violence, and the pressure of administration all combine to bring about an overall feeling of professional burnout. Addressing burnout therefore requires structural reforms, including safe patient-load limits, strengthened workplace safety policies, adequate staffing ratios, and equitable income structures, in addition to individual-level psychological support interventions. There is an immediate need of institutional changes that will take into consideration the safety of workplaces, fair remuneration, and the sufficiency of staffing. Psychological interventions aimed at mental health care, regulation of work, and restoration of work-life balance should be taken as the main measures to guarantee the well-being of physicians and sustainability of healthcare provision in Türkiye.

## 5. Conclusion

This study reveals that a substantial proportion of physicians and dentists working in Türkiye experience moderate to high levels of burnout. Major contributors include challenging working conditions, excessive workload, lack of nonphysician support staff, low perceived income, shift-based work, exposure to violence, and mobbing. The findings indicate that reducing burnout among physicians and dentists would not only improve physician and dentist satisfaction but also positively impact the healthcare system. Physicians and dentists with low burnout and high job satisfaction can provide more effective and higher-quality care, thus contributing to the improvement of the overall health system.

Preventing physician and dentist burnout will strengthen doctor–patient relationships, improve healthcare service quality, and move the system toward a more sustainable and ideal model. Therefore, it is crucial to improve working conditions, enhance support mechanisms, and rebalance workloads to increase professional satisfaction and prevent burnout. In practical terms, this may include implementing structured workplace violence prevention programs with zero-tolerance policies and rapid legal response mechanisms, setting institutional caps on daily outpatient volume (e.g., limiting routine outpatient visits to a maximum of 40 patients per physician per day to reduce cognitive overload and time pressure), and establishing staffing adequacy standards. Increasing the number of nonphysician healthcare personnel (e.g., nurses and allied health professionals) to levels at least meeting or exceeding the OECD average may help distribute workload more equitably and reduce administrative and clinical burden on physicians. Additionally, strengthening institutional legal protections, ensuring transparent and performance-aligned income structures, and promoting organizational policies that safeguard work–life balance are critical steps toward mitigating systemic contributors to burnout. By reducing burnout, we can achieve happier physicians and dentists, more satisfied patients, and a more resilient healthcare system. This study serves as a significant reference point for future health policies and initiatives aimed at enhancing the professional well-being of physicians and dentists.

Although the findings reflect a large and heterogeneous national sample, generalizability to other countries should be interpreted cautiously, given differences in healthcare systems, cultural contexts, and policy environments. Future longitudinal and multinational studies are needed to clarify causal pathways and assess cross-system applicability.

*Limitations*: The current study appreciates the fact that there are the following limitations which should be recognized. The first one is that the cross-sectional design does not allow us to build causal relationships and thus we can just report associations and not causal relations. Second, the use of online self-reported questionnaires also opens the risk of response bias because the respondent will be tempted to under- or overreport some experiences because of personal or professional sensitivities. Third, the distribution of the questionnaire via online platforms, including social media and professional networks, may have introduced selection bias, as physicians who are less active on digital platforms might have been underrepresented. This limitation may restrict the generalizability of the findings. Forth, internal reliability coefficients (e.g., Cronbach alpha) were not recalculated in this dataset, which may limit evaluation of internal consistency within the current sample. Lastly, convenience sampling as opposed to random sampling can be a limiting factor to the applicability of the results to the entire population of physicians in Türkiye.

## Acknowledgments

The authors would like to thank all participating physicians and dentists for their valuable time and contributions to this nationwide survey. We also acknowledge the support of professional physician and dentist networks and associations that facilitated the distribution of the study questionnaire. The authors also thank Dr Önder Aydemir, Dr Erkut Etçioğlu, Dr Ali Muhtaroğlu, Dr Kubilay İşsever, and Dr Ahmed Cihad Genç for their valuable contributions. The individuals named in the acknowledgments section have provided permission to be acknowledged.

## Author contributions

**Conceptualization:** Mehmet Yildiz, Abdülkadir Aydin.

**Data curation:** Mehmet Yildiz, Muhammet Raşit Aydin.

**Formal analysis:** Mehmet Yildiz, Muhammet Raşit Aydin.

**Funding acquisition:** Mehmet Yildiz.

**Investigation:** Mehmet Yildiz, Abdülkadir Aydin.

**Methodology:** Mehmet Yildiz.

**Project administration:** Mehmet Yildiz.

**Resources:** Mehmet Yildiz, Muhammet Raşit Aydin.

**Software:** Mehmet Yildiz, Abdülkadir Aydin.

**Supervision:** Mehmet Yildiz, Muhammet Raşit Aydin, Abdülkadir Aydin.

**Validation:** Mehmet Yildiz, Muhammet Raşit Aydin, Abdülkadir Aydin.

**Visualization:** Mehmet Yildiz, Abdülkadir Aydin.

**Writing – original draft:** Mehmet Yildiz.

**Writing – review & editing:** Mehmet Yildiz.
